# Updates to the antitumor mechanism of oncolytic virus

**DOI:** 10.1111/1759-7714.13043

**Published:** 2019-03-22

**Authors:** Yang Bai, Peng Hui, Xiaoyu Du, Xing Su

**Affiliations:** ^1^ Department of Neurosurgery The First Hospital of Jilin University Changchun China; ^2^ Department of Ophthalmology The First Hospital of Jilin University Changchun China; ^3^ Department of Cardiovascular Center The First Hospital of Jilin University Changchun China; ^4^ The Laboratory of Cancer Precision Medicine The First Hospital of Jilin University Changchun China

**Keywords:** Clinical study, oncolytic mechanism, oncolytic virus

## Abstract

Oncolytic viruses (OVs) are promising new therapeutic agents in the field of malignant tumor treatment. OVs can achieve the goal of targeted therapy by selectively killing tumor cells and inducing specific antitumor immunity. The key roles of OVs are tumor targeting and tumor killing mechanisms. Recently, molecular biotechnology has been used to optimize the transformation of wild virus strains in order to ensure a stronger oncolytic effect and lower adverse reactions, to enable testing in clinical trials as an antitumor drug. The main purpose of this review is to provide a description of oncolytic mechanisms, clinical studies, combination therapies, current challenges, and future prospects of OVs.

## Introduction

The global rate of cancer incidence has risen in recent years.^1^ Cancer is still a significant factor threatening human health. Cancer cells use high mutation to evade immune surveillance, gradually forming a low pH, low oxygen microenvironment containing proteolytic enzymes.^2^ In addition, cancer cells can undergo infinite replication and metastasis, ultimately leading to organ failure and patient death.^3^ With continuous advancements in treatment methods, studies have found that the virus has great potential for cancer treatment. Oncolytic viruses (OVs) are able to target and kill tumor cells by selecting some strains in nature with weak pathogenicity, and certain viruses can be genetically modified.^4^

The antitumor potential of OVs has been shown in various historical studies indicating that viral infections alleviate the original tumor symptoms.^5^ For example, PVSRIPO, the type 1 (Sabin) live‐attenuated poliovirus vaccine replicating under the control of a heterologous internal ribosomal entry site of human rhinovirus type 2, has shown early promise in phase I clinical trials of recurrent glioblastoma patients.^6^ It has been reported that clinical symptoms in patients with blood diseases or lymphoma have been relieved after infection with varicella virus, measles, and hepatitis B virus.^7^ As a result, exploration of the specific mechanism of viral antitumor continues. Generally, a virus will be cleared by the immune response upon entering the body. Although some patients develop syndromes related to viral infection, tumor symptoms continue to decrease. Currently, there are dozens of OVs for cancer treatment, including adenovirus, herpes simplex virus, Newcastle disease, vaccinia virus, reovirus, and vesicular stomatitis virus.^8^ This article provides an overview of the oncolytic mechanisms, research progress, and clinical application of OVs.

## Classification of oncolytic virus

OVs can be divided into two major categories according to development: natural viruses and genetically modified virus strains. Natural viruses include wild type and naturally variant strains of weak viruses. For example, reovirus is a wild‐type OV that only replicates in cells with an activated Ras signaling pathway and specifically targets Ras‐activated cancer cells. Reovirus can target *EGFR* overexpressed tumor cells. *EGFR* activates the Ras signaling pathway to produce a phospholipase that antagonizes double‐stranded RNA‐dependent protein kinases, thereby promoting OV replication.^9^ NDV is a kind of RNA virus. In normal cells, single‐stranded RNA carried by NDV replicates to form double‐stranded RNA, which induces high expression of protein kinase PKR and activates the interferon signaling pathway, causing an antiviral response. However, as a result of defection of the interferon signaling pathway in cancer cells, there is no antiviral effect, and NDV survives replication in tumor cells.^10^ NDV‐HUJ is a type of attenuated virus strain of NDV that has been used in clinical studies of recurrent glioblastoma multiforme.^11^ Although wild attenuated strains have lower viral toxicity and better tumor killing effects than other viruses, their clinical application is unsatisfactory. With the development of molecular biology techniques, genetic editing technology has been used to optimize these wild virus strains, for example, to weaken viral pathogenicity and improve immunogenicity.^12^ Insertion of an exogenous therapeutic gene into the OV genome, to increase its expression in the tumor, makes it is possible to avoid the occurrence of a systemic immune response and enhances the lethality of the virus. A virus strain can be selected as a genetically modified strain according to weak virus toxicity or targeted tumorigenic properties. Present studies are focusing on HSV‐1 and adenovirus strains. The ability of HSV‐1 killer cells is inherent, infecting a wide variety of cells and lysing cells through continuous self‐replication, and as its genome contains many non‐essential genes, it is more easily genetically modified. A variety of oncolytic viruses based on HSV‐1 have been widely used in cancer therapy. The most noteworthy T‐Vec has completed phase III clinical trials in advanced melanoma patients and achieved significant therapeutic effects.^13^ Adenovirus is a non‐enveloped double‐stranded DNA virus with relatively small genomes, which is easy to genetically modify, prepare, and purify. Therefore, the adenovirus has been the most commonly researched virus in recent studies. Because of the shortcomings of the virus, such as relatively low replication capacity, poor specificity, and the hepatotoxicity of the virus itself, the clinical efficacy of adenovirus is not significant.

## Mechanisms of oncolytic virus action

OV treatment refers to the use of a virus to self‐replicate to destroy the host cells in infected cancer cells. By hijacking the cell's protein synthesis, the virus prevents the cell from producing host products and promotes the production of viral products. The infected host cells will lyse and release many subviruses that have the ability to infect other cells.^14^ The growth of the tumor must inhibit the immune system; however, immune suppression not only has systemic effects, but also is related to a tumor's internal environment, as cancer cells secrete some factors that regulate the immune cells to escape immune surveillance. For example, non‐specific immune cell macrophages have changed from an aggressive M1 type to a conservation‐type M2 type, which not only not kills tumor cells, but also induces angiogenesis and secretes many growth factors to nourish tumors.^15^ In recent years, the vigorous development of tumor immunotherapy has used a passive method to relieve tumor‐induced immunosuppression.^16^, ^17^ The immune response caused by an OV infecting the body can be summarized as follows. The virus has a strong stimulating effect on immune cells infiltrating the tumor tissue, which can greatly alter the tumor microenvironment. Virus‐infected tumor cells are able to express so‐called “danger signals” (such as cytokines), which can induce immune cells outside the tumor to infiltrate the tumor and activate non‐specific immune cells. Tumor cells lysed by OV release large amounts of tumor proteins that can be phagocytized by non‐specific immune cells, and certain tumor‐specific antigens can be expressed by these antigen presenting cells, inducing T cells to attack uninfected tumor cells. A virus can be constructed with natural infection ability and increased immunogenicity by knocking out, inserting, or transferring a foreign gene. Many offspring of OVs have been constructed to express a foreign gene and produce a specific cytokine (such as human TNF, granulocyte‐macrophage colony‐stimulating factor, IL‐7, IL‐12, IFN‐β, etc.).^13^, ^18^, ^19^ This method of allowing a virus to express a foreign gene is like an “armed” OV that enhances the ability of cell lysis.

In order to improve the targeting ability of a virus, viral medication methods should include the following aspects. Choose natural viruses that specifically infect tumors, especially host cells with tumor‐associated mutations, including reovirus, Newcastle disease, parvovirus, varicella virus, and sindbis virus;^20^ other viruses may be involved in the tumor microenvironment and tumor stroma (including herpes virus, measles virus). Mutation and/or lack of certain virus genes: first‐generation OVs are single‐gene deletions, while second‐generation OVs are polygenic deletions, all of which play an important role in the replication of the virus in normal cells; however, these genes are insignificant to replicate the virus in tumor cells^21^ Put the key genes of the virus under tumor‐specific and/or tissue‐specific gene promoters, so that the expression of key genes of the virus can be restricted in tumor cells, thereby improving safety; for example, human telomeres enzyme reverse transcriptase promoter^22^ Human telomerase reverse transcriptase is highly expressed in tumor cells but is not expressed or is underexpressed in normal cells, thus increasing the tumor targeting ability of the virus and altering the tropism of the virus, allowing the virus to bind only to specific receptors on the surface of tumor cells, such as the adenovirus Delta‐24RGD. Herpes simplex virus envelope glycoprotein D binds to the cell surface receptor‐binding protein 1 with high affinity.^23^

The interaction between tumor cells and the tumor microenvironment is important to the occurrence and development of tumors. The formation of the tumor microenvironment may hinder the efficacy of an OV. In order to eliminate the unfavorable factors caused by the tumor microenvironment, the OV may be assisted by drugs or genetic modification to promote efficacy. The extracellular matrix (ECM) in solid tumors can affect the infection and spread of therapeutic viruses. Matrix degrading enzymes can improve the permeability of tumors by degrading the ECM, thereby enhancing the ability of the virus to spread, and increasing the concentration of the virus in tumor cells.^24^ The tumor microenvironment has a regulatory effect on the activation of the innate immune response. Environmental changes can lead to the rapid removal of the virus and limit its efficacy. Studies have shown that viral combined immunosuppressive agents, such as sunitinib, can form an immunosuppressive microenvironment in tumors by limiting natural immune systems, such as protein kinase PKR and 2′‐5′ oligoadenylate synthetase, attenuating the antiviral innate immune response and enhancing the antitumor effect of the virus.^25^ The combination of vesicular stomatitis virus and sunitinib in tumor‐bearing mice can significantly inhibit the growth of malignant tumors, such as prostate, breast, and kidney cancers.^26^

In the process of OV treatment, the neutralizing antibody with antiviral effect restricts the proliferation and spread of the virus via natural immunity. On the other hand, the virus can stimulate the body to produce adaptive immune killing tumor cells by mimicking the immune co‐stimulatory molecule. The immune system plays an important role as an inhibitory or stimulating factor in the treatment of OVs. When a virus enters the body, it is usually recognized as a pathogen invasion, and an immune response is then initiated. This immune response system includes signal transduction pattern recognition receptors and pathogen‐associated molecular patterns induced by selective or specific signal transduction pathways, such as PKR, p53, and phosphorylated retinoblastoma protein pathways.^27^ Such signal transduction pathways in tumor cells are often abnormal, leading to the replication and amplification of the virus in such tumor cells.^28^ The efficacy of OV treatment depends on the ability of the virus to replicate and spread. Therefore, an urgent problem is how to prevent the virus being bound by the innate immune response before it can target the tumor cells. Studies have shown that the way in which the virus enters the body affects the strength of the host's antiviral response, which in turn affects the oncolysis of the virus. General methods of administration (e.g. intravenous and oral), cause an innate immune reaction whereby the antibody neutralizes the virus before it targets the tumor; thus the oncolytic effect cannot be exerted.^29^ It is possible to use antiviral reactions, such as histone deacetylase inhibitors and immunomodulatory drugs, along with the natural reaction to eliminate viral mechanisms, but a large number of experiments are required to verify the application of such drugs.^30^ After the virus enters the tumor cells, the virus‐infected tumor cells can further express cytokines or directly affect the immune cells, activate the body's immune system to kill and phagocytose residual tumor cells. The antitumor immune response of the body is enhanced by the recombination of OVs and immunostimulatory molecules.

## Delivery of oncolytic virus

Oral viral vaccines are suitable for poliovirus, rotavirus, rabies virus, typhoid virus, etc.,^31^ but the immunity of the oral route is weaker than the parenteral route. Many viruses are inactive as a result of the pH in the gastrointestinal tract and multiple enzymes. The intravenous route is a convenient route of administration, but there are a number of drawbacks. Intravascular administration does not specifically target cancer. In addition, the antibodies produced by the pre‐existing immunity will neutralize the virus before it reaches the tumor. It is necessary to increase the virus concentration to the target site, but this may increase the inflammatory response. Further research is required to improve the targeting ability of a virus to reduce the virulence to the venous system and increase oncolytic efficiency.^28^ Two routes of radiation intervention are arterial and tumor site administration. Other possible routes include the portal vein, intracoronary, and thoracic medication. The intra‐arterial approach has many advantages. The virus can be selectively transported to target cells. The time of drug retention is related to embolic material and the dilatation balloon. Because of the limited volume of blood and the target organ, this method can effectively avoid antibody neutralization. Administration to the tumor site is similar to percutaneous ethanol injection; however, it is not easy to control and monitor the distribution of a virus via this method.

When the infected tumor tissue becomes necrotic and dissolves, the virus is released and will continue to infect adjacent uninfected tumor cells, releasing the virus into the blood, which may become viremia or a distant infection.^32^ Another method of delivering the virus into the body has not been applied in clinical trials, but it has been widely considered for a long time; that is, using the patient's cells as a viral vector. The strategy uses an autologous cell that is inherently tumor‐oriented to act as a viral vector, particularly on immune cells.^33^ It is expected that cells carry the OV to the distant malignant tumor.^34^ This method has been demonstrated in many animal models.^35^ Many cells have become candidates for viral vectors, but further experiments are required for confirmation, such as tumor infiltrating lymphocytes or mesenchymal stem cells.^34^ In the early stage of clinical trials, autologous adipose‐derived mesenchymal stem cells could serve as viral vectors and have gained certification from the United States Food and Drug Administration.^36^ The mesenchymal stem cells from the adipose tissues of healthy donors and nine ovarian cancer patients were characterized for susceptibility to virus infection and tumor‐homing abilities.

## Immune therapy and oncolytic virus combinations

The combination of immunotherapy drugs and OV treatment is a potential direction for future cancer treatment. The PD‐1/PD‐L1 blocker is currently a popular antitumor drug. The PD1/PD‐L1 blocker is mainly used to block the inhibition of T cells by tumor cells, thereby activating the immune system to attack tumor cells.^37^ However, in tumors with no or low levels of PD‐L1 expression, the blocker has no inhibitory site and therefore cannot exert antitumor effects.^38^ When s PD‐1/PD‐L1 blocker is combined with s virus, the OV infects the tumor cells and induces a large number of immune cells (T cells) to infiltrate the tumor. In the face of T cell invasion, the tumor cells will further increase the PD‐L1 expression level, generating a self‐protection mechanism to escape the immune attack, which in turn expands the scope of the blocker. Liu *et al.* confirmed that the combination of the vaccinia virus and a PD‐L1 antibody was better than single application in a colon cancer xenograft model.^39^

## Conclusion

In summary, after continuous exploration and research, the mechanism of OV for killing tumors has become increasingly clear, and clinical trials are gradually unfolding. OVs have immeasurable application potential and market as a treatment method, but there are still some defects in the process of clinical application. The tumor microenvironment is very complicated. Although current molecular biotechnology has enhanced the targeting and killing effects of OVs, more extensive basic research and clinical trials are required to pursue more effective and less adverse tumor treatment.

## Disclosure

No authors report any conflict of interest.
